# Stroke in COVID-19 and Pregnancy: A Case Report

**DOI:** 10.1590/0037-8682-0301-2021

**Published:** 2021-08-20

**Authors:** Mylana Dandara Pereira Gama, José Ronaldo Lessa Angelo, Carolina da Cunha-Correia

**Affiliations:** 1 Universidade de Pernambuco, Hospital Universitário Oswaldo Cruz, Recife, PE, Brasil.; 2 Universidade de Pernambuco, Faculdade de Ciências Médicas, Departamento de Neurologia, Recife, PE, Brasil.

**Keywords:** Coronavirus infections, Pregnancy, Cerebrovascular accident

## Abstract

Neurological manifestations add prognostic severity to the coronavirus disease (COVID-19). Here, we report a case of a pregnant patient with COVID-19 that progressed with neurological complications. Magnetic resonance imaging revealed cerebral ischemic insults associated with cortical laminar necrosis, in addition to an intraparenchymal brain hematoma. The mechanisms of vascular injury may have multifactorial origins and result in complex radiological presentations. Since stroke associated with pregnancy is one of the main causes of long-term disability in women, accurate identification of cerebrovascular events may potentially reduce sequelae.

## INTRODUCTION

COVID-19, a disease caused by a new respiratory virus called severe acute respiratory syndrome coronavirus 2 (SARS-CoV-2), typically presents as an acute febrile disease, with respiratory involvement as its most common form^1^. Additionally, viruses in the *Coronaviridae* family can cause neurological affection^2^. In April 2020, the first observational study of acute complications of COVID-19 found neurological manifestations in 36.4% of the hospitalized patients, 24.8% of which were central, and 8.9% were peripheral nervous system manifestations^1^. Since then, descriptions have become frequent. The first series of cases of cerebrovascular accident (CVA) associated with COVID-19 was published in April 2020, and the first case report of cerebral venous thrombosis (CVT) in May of the same year^2^.

Even before the emergence of the new coronavirus, studies have reported that recent respiratory tract infections increase the short-term risk of CVA^3^. The proportion of patients infected with COVID-19, who are expected to have a CVA is around 4.9% (95% confidence interval: 2.8-8.7%); however, more robust studies are needed. COVID-19-related hemorrhagic strokes are much less common than ischemic events^4^. 

From the epidemiological perspective, and since CVA associated with pregnancy is the main cause of severe long-term disability in women of childbearing age^5^, the objective of this article is to describe a CVA case in a pregnant COVID-19 patient, with the aim of discussing the peculiarities of neuroimaging findings and possible factors associated with distinct cerebral vascular events in a single patient. 

## CASE REPORT

A 34-year-old woman, 26 weeks pregnant, was admitted to a hospital in Recife-PE, in April 2020, with persistent headache associated with respiratory symptoms, which progressed with a drop in oxygen saturation and the need for orotracheal intubation after 5 days of admission.

As the patient was in a critical condition, pregnancy termination was carried out, with subsequent fetal death. Her condition worsened in the intensive care unit, with hyperactive delirium and focal seizure, characterized by blink and left upper limb clonic movement with progression to generalization. Neurological examination revealed left hyperreflexia and left homonymous hemianopsia.

During the investigation, the presence of SARS-COV-2 in the nasal swab was confirmed and analyzed using *real*-*time polymerase chain reaction*. Serum analysis revealed an increase in D-dimer (3,504 μg/L) levels, leukopenia (2,850 K/µL), mild anemia (10.6 g/dL), hematocrit of 31.5%, and thrombocytopenia (136,000/mm3). High C-reactive protein (26.68 mg/dL) levels and erythrocyte sedimentation rate (45 mm/h). Normal levels of serum iron, ferritin, troponin, total *creatine kinase* and *creatine kinase* MB levels. 

Chest computed tomography with contrast showed an interstitial pattern in ground-glass opacity in both lungs. Transthoracic echocardiography showed moderate reduction in left ventricular systolic function, suggesting myocarditis. 

**FIGURE 1:**
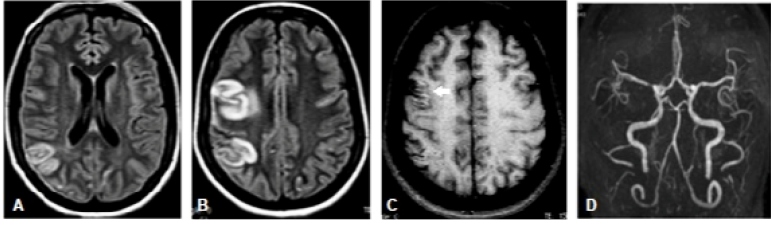
MRI. Axial T2 FLAIR weighted images (**A, B**). T1-weighted axial images without venous contrast (**C**) and arterial 3D TOF MRA (**D**). We noticed hypersignal on T2/FLAIR at the right frontoparietal lobes , with spinning thickening and mild vasogenic edema, corresponding to ischemic arterial vascular injury. There is also cortical spontaneous hypersignal on T1, without expression on T2* images, related to cortical laminar necrosis. There is no mass or reduction on the ADC map on these changes (not shown). We noticed slight signal loss in the proximal segment (M1) of the right middle cerebral artery, in addition to clear asymmetry of its distal segments (M3), with mild vascular poverty to the right (**D**). **MRI**, magnetic resonance imaging; **FLAIR**, fluid-attenuated inversion recovery; **MRA**, magnetic resonance angiography; **3D TOF**, three-dimensional *time of flight;* **ADC**, apparent diffusion coefficient

Magnetic resonance imaging of the brain showed signal hyperintensity on T2 FLAIR at the right frontoparietal lobes, related to ischemic arterial vascular event (Figure 1. A-C); while the magnetic resonance angiography (MRA) without venous contrast revealed asymmetry of the middle cerebral arteries, with subtle changes to the right (Figure 1. D). 

**FIGURE 2:**
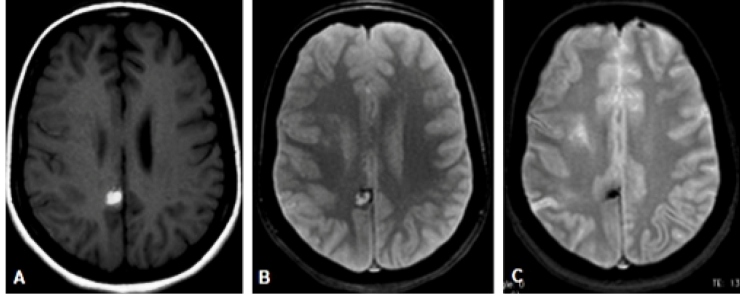
MRI. T1-weighted axial images (**A**) and T2*-weighted axial images (**B** and **C**). A posterior rounded lesion in in the cingulate gyrus, with spontaneous hypersignal on T1 (**A**) and hyper with hypointense halo on T2* (**B**), related to parenchymal hematoma. Control examination shows hematoma in the chronic stage, with reduction in size and marked hypointensity on T2* related to hemosiderin (**C**). **MRI**: Magnetic resonance imaging; **FLAIR**: fluid-attenuated inversion recovery

Additionally, we noticed a posterior rounded lesion in the cingulate gyrus, with spontaneous hypersignal on T1 (Figure 2. A) and hypersignal with a hypointense halo on T2* (Figure 2. B), which was related to a parenchymal hematoma (Figure 2. A-C).

Hypercoagulable studies were normal, including antinuclear antibody, lupus anticoagulant, anti-cardiolipin, anti-dsDNA, perinuclear antineutrophil cytoplasmic antibodies, and antineutrophil cytoplasmicantibody levels.

The patient received oral anticoagulants and oxcarbazepine to control the seizures. She was discharged after 45 days, with good clinical recovery and a Mini-Mental State Examination score of 29 points.

This study was approved by the ethics committee of Oswaldo Cruz University Hospital, Certificate of Presentation for Ethical Consideration (CAAE) number 34779420.1.0000.5192, and consent letter from Unimed I Hospital.

## DISCUSSION

SARS-CoV-2 is the seventh known variant of coronavirus that infects humans. It is genetically similar to SARS-CoV-1, which affected approximately 8,000 patients in the year 2003, with some reports of neurological manifestations, mainly peripheral neuropathy and encephalitis. Currently, reports and case series have associated SARS-CoV-2 with manifestations, such as cerebrovascular events, peripheral disorders, posterior reversible encephalopathy syndrome (PRES), acute necrotizing encephalopathy, and psychiatric disorders^2^. 

A U.S. case-control study demonstrated that COVID-19 is an independent risk factor for ischemic CVA, suggesting that viral infection is associated with an increase in morbidity and mortality, which transcends the primary cardiopulmonary sequela of the infection^3^. We may consider the incidence of ischemic CVA from 0.9% to 2.7%^8^, or up to 4.9% in other studies^6^, with a median time between disease onset and CVA symptoms of 10 ± 8 days, and prevalence of large vessel occlusion of 40.9%^8^.

Several theories link infectious-inflammatory syndromes to an increased risk of CVA. The virus affinity for angiotensin-converting enzyme (ACE) 2 receptors, which are expressed in the endothelium and arterial smooth muscle cells in the brain, seems to elicit an inflammatory response that is believed to be a possible basis for thrombotic complications. There also seems to be vessel involvement by the systemic immune response to the pathogen, known as a cytokine storm^6^ ^-^ ^8^. Other vessel-mediated mechanisms, including altered vasomotor reactivity, may play a role in neurological symptoms, since it is known that SARS-CoV-2 may disrupt the renin-angiotensin-aldosterone system^6^ ^-^ ^9^.

As for the evidence of coagulopathy, a 4 times above normal level of D-dimer is associated with a 5-fold increase in the probability of critical disease^6^, and there may also be increased serum fibrinogen levels (94% of patients), platelets (62%), and interleukin-6 (62%)^2^. Such data are compatible with the presentation of the patient, in which the D-dimer level reached 7 times the normal level, and her progression required orotracheal intubation. In critically ill patients, COVID-19 has been associated with coagulopathies that may result in hemorrhage, since SARS-CoV-2 may induce ACE2 downregulation*.* This event may lead to vasoconstriction and dysfunction of cerebral self-regulation, with consequent pressure peaks that may lead to rupture of the arterial wall and eventually cause hemorrhage^2^.

The patient progressed with respiratory failure and myocardial dysfunction documented by ultrasonography, with neuroimaging, which suggests that the insults might have occurred from embolization, originating in branches of the right middle cerebral artery. Another important aspect is the presence of suggestive images of focal laminar cortical lesions^10^. We suspect that this pattern might be related to a possible transient dysregulation of vasomotor reactivity, whose cortical involvement may suggest a possible vascular mechanism associated with transient vasoconstriction. Such involvement of cortical prevalence and its localization are not typical of PRES, and the hypothesis of cortical embolic infarctions is unlikely^10^. A neuroimaging study including 103 COVID-19 patients reported that most ischemic strokes occur due to thrombosis, embolism, or stenosis of the great vessels pattern (62.1%) followed by the pattern of multiple vascular territories (26.2%) and then infrequent small vessel patterns (8.7%)^8^. The peculiarity of the laminar cortical lesion alerts the hypoperfusion component. 

A systematic review of current scientific literature to identify the evidence of intracranial hemorrhage in COVID-19 patients demonstrated that intraparenchymal hemorrhage was the most common pattern (62.6%), followed by subarachnoid hemorrhage (15%), subdural hemorrhage (11.6%), and intraventricular hemorrhage (1.4%). Of these, the majority (93.5%) were lobar^11^. In this case report, in addition to the anticoagulant indicated due to the severe viral infection, the patient was in the postpartum period, and therefore, was prone to thrombohemorrhagic events. We also considered the possibility of a brain parenchymal hematoma resulting from venous thrombosis of a small cortical vein, although we did not spot the thrombosed vein adjacent to the referred hematoma in the venous phase of MRA (not shown).

The incidence of CVA during pregnancy may be estimated to be at 16.6/100,000 deliveries, with a higher occurrence as gestational age progresses. Pregnancy-associated CVA is cited as the leading cause of severe long-term disability in postpartum women. It is estimated that 58.6% of the cases are hemorrhagic, 28.4% ischemic, 4.2% CVT, and 9.4% stroke not specified, with a mortality of 7.4% and a higher incidence in the puerperium^5^. Little has been published on neurological complications in pregnant women infected by SARS-CoV-2, but studies suggest that a subgroup might progress with multiple organ failure or death, in addition to a higher risk of thrombotic events^5^ ^,^ ^9^ ^,^ ^12^. 

Although the patient was young, she presented with an arterial event, and it should be noted that brain vascular events occur mainly in pregnant women over 35 years of age^5^ and in elderly patients infected with SARS-CoV-2^2^. We warn of the possibility of arterial ischemia associated with CVT in the occurrence of an intraparenchymal hematoma. The report of this case is justified by the complexity of clinical and neuroimaging manifestations with which severe COVID-19 patients may present, with the potential for higher morbidity in pregnant patients.

In conclusion, the actual incidence of CVA in patients with COVID-19 has not yet been well established. The risk of these vascular events may be increased in patients with severe forms of the disease. Clinical-radiological presentations may be variable and require the exclusion of autoimmune thrombophilia in pregnant patients.
